# Feasibility and Acceptability of a Multilayered COVID-19 Mitigation Intervention for Adults With Cancer

**DOI:** 10.1001/jamanetworkopen.2025.60547

**Published:** 2026-02-24

**Authors:** Michael Hoerger, Nicole Pyke, Danielle E. Zimmerman, Devabhaktuni Srikrishna, Nicole Garg, Brooke Mikles, Joseph Eastman, Caroline J. Hall, Rebecca G. Peter, James I. Gerhart

**Affiliations:** 1 Cancer Population Science, Tulane Cancer Center, Tulane University, New Orleans, Louisiana; 2 Department of Psychology, Tulane University, New Orleans, Louisiana; 3 The Ohio State University Comprehensive Cancer Center–Arthur G. James Cancer Hospital and Richard J. Solove Research Institute, Columbus; 4 Patient Knowhow, San Mateo, California; 5 Department of Social Work, Columbia University, New York, New York; 6 Department of Psychology, Tulane University, New Orleans, Louisiana; 7 World Health Network, Science and Policy Group, Bovey, Minnesota; 8 School of Public Health, Tulane University, New Orleans, Louisiana; 9 Lopez, Washington; 10 Department of Psychology, Ohio University, Athens

## Abstract

This quality improvement study evaluated the feasibility, acceptability, and cost of a multicomponent, multilayered COVID-19 mitigation toolkit for adults with cancer.

## Introduction

Multifaceted interventions may help patients with cancer avoid severe COVID-19 outcomes. The SARS-CoV-2 virus that causes COVID-19 transmits through 2 waves annually in the US.^1^ COVID-19 may have caused a 6% increase in excess deaths in the US and United Kingdom in 2025, equivalent to more than 200 000 deaths, predominantly among high-risk individuals.^2^ The eDelphi Study to Fully Define and COVID-Risk Stratify Immunosuppression (DESTINIES) international consensus study identified patients undergoing cancer treatment as the largest population at high risk.^3^ People with cancer experience a diminished vaccine response and have elevated risk of COVID-19–associated hospitalizations, long COVID, and COVID-19–related mortality.^3,4^ Infections often mean halting cancer therapy.^5^ Leading cancer centers increase precautions during waves.^6^ However, patients and families must navigate daily risks with limited guidance. We developed a mailed, multicomponent COVID-19 Defense Toolkit grounded in multilayered mitigation^4^ and conducted a single-arm pilot study to evaluate intervention feasibility, acceptability, and cost for adults with cancer.

## Methods

In this quality improvement study, we recruited adult patients receiving cancer therapy at an academic cancer center and through nationwide remote outreach from February 24 to September 28, 2024 (eMethods in Supplement 1). Eligible participants were 18 years or older, living in the US, and undergoing active cancer treatment. The study was reviewed and approved by the Institutional Review Board of Tulane University and adhered to the SQUIRE reporting guideline. After completing an introductory call, an electronic informed consent form, and a baseline assessment, participants were mailed an individually tailored toolkit containing an illustrated educational booklet on risk reduction, 50 high-filtration N95/KN95/KF94 masks selected to meet family needs, 2 portable air purifiers, and 5 rapid antigen tests. Participants completed a 1-month follow-up survey about utilization, attitudes, and perceived safety. Prespecified feasibility thresholds were enrollment of at least 30 participants, successful delivery of at least 90% of toolkits, and at least 70% retention at 1 month. The intervention was considered acceptable if mean scores were 7 or greater on 5 ratings of 0 to 10 assessing perceived safety and likelihood of recommending each component. Microcosting analyses estimated toolkit and maintenance costs. Analyses included descriptive statistics (proportions, means, and SDs), powered considering 95% CIs and 2-sided α = .05 (eMethods in Supplement 1).

## Results

We contacted 37 patients, 32 of whom were enrolled; 1 died while their toolkit was in transit, leaving 31 who were included in the final analysis. The mean (SD) age was 58.4 (13.2) years; 19 patients (61.3%) were female and 12 (38.7%) were male; and 27 patients (73.0%) had less than a college degree (Table 1).

**Table 1.** Patient Characteristics

Characteristic	No. (%) of patients (N = 31)
Age, mean (SD), y	58.4 (13.2)
Sex	
Female	19 (61.3)
Male	12 (38.7)
Race and ethnicity^a^	
Asian	1 (3.2)
Black or African American	10 (32.3)
Hispanic or Latino or Latina	3 (9.7)
White	23 (74.2)
Other^b^	0
Bachelor’s degree or higher educational level	10 (32.3)
Married or living with a partner	20 (64.5)
Employed	13 (41.9)
Financial status	
Very financially strained	8 (25.8)
Lower middle class	3 (9.7)
Middle class	16 (51.6)
Upper middle class	4 (12.9)
Very well off	0
Location	
Local, New Orleans metro region	15 (48.4)
Regional, Louisiana	8 (25.8)
National, US	8 (25.8)
Current health	
Poor	5 (16.1)
Fair	16 (51.6)
Good	9 (29.0)
Very good	1 (3.2)
Excellent	0
Cancer site	
Hematologic	9 (29.0)
Breast	7 (22.6)
Gynecologic	4 (12.9)
Skin	4 (12.9)
Genitourinary	3 (9.7)
Gastrointestinal	2 (6.5)
Lung	2 (6.5)
Treatments	
Chemotherapy	19 (61.3)
Immunotherapy	19 (61.3)
Surgery	14 (45.2)
Radiotherapy	13 (41.9)
Hormonal therapy	5 (16.1)
Stem cell transplant	1 (3.2)
Time since diagnosis <1 y	20 (64.5)
No. of comorbidities, mean (SD)	2.00 (1.67)
Known positive history of COVID-19	18 (58.1)
Baseline precautions	
Vaccinated in the past year	11 (35.5)
Receipt of COVID-19 print information in cancer care	3 (9.7)
N95 mask use ever	24 (77.4)
Rapid test use ever	29 (93.5)
In-home air purifier use ever	13 (41.9)

^a^ Self-reported totals exceed 100% because some participants reported more than 1 race or ethnicity.

^b^ Includes American Indian or Alaska Native and Native Hawaiian or Other Pacific Islander.

All feasibility and acceptability criteria were exceeded. All toolkits were delivered successfully, 31 participants (100%) completed the 1-month follow-up survey, and the project’s high demand yielded a waiting list of 366 potentially eligible patients. The toolkit met 100% of 5 core acceptability criteria (Table 2). Patients gave high mean (SD) ratings for toolkit safety (8.5 [1.7]) and likelihood of recommending the booklet (9.4 [1.0]), masks (9.9 [0.5]), air purifiers (9.6 [0.9]), and tests (9.8 [0.6]) to other patients. Nearly all participants reported using the booklet (30 [96.8%]), masks (30 [96.8%]), and air purifiers (29 [93.5%]); 16 (51.6%) used rapid tests. The toolkit landed cost was $219 per participant; based on utilization levels, estimated maintenance costs were $0.99 per day.

**Table 2.** Acceptability of the COVID-19 Defense Toolkit^a^

Acceptability outcome	Values
Toolkit	
Provided safety from COVID-19, mean (SD) score	8.5 (1.7)^b^
Helped deal more effectively with COVID-19 risk, No. (%)	31 (100)
Helped deal more effectively with COVID-19–related stress, No. (%)	30 (96.8)
Overall satisfied or very satisfied, No. (%)	27 (87.1)
Likely or very likely to accept the toolkit if offered again, No. (%)	30 (96.8)
Likely or extremely likely to recommend to others with cancer, No. (%)	30 (96.8)
Educational booklet	
Recommended to others with cancer, mean (SD) score	9.4 (1.0)^b^
Read personally or by family, No. (%)	30 (96.8)
No. of times opening the booklet, mean (SD)	3.8 (3.4)
Satisfied or very satisfied with length and depth, No. (%)	29 (93.5)
No difficulty reading the booklet, No. (%)	30 (96.8)
Rated in the top 2 of the 4 toolkit components	9 (29.0)
Agree or strongly agree reading was worthwhile, No. (%)	29 (93.5)
Masks	
Pack recommended to others with cancer, mean (SD) score	9.9 (0.5)^b^
Total No. of masks used (0-50), mean (SD)	16.9 (12.7)
Any use, No. (%)	30 (96.8)
Rated in the top 2 of the 4 toolkit components, No. (%)	22 (71.0)
Protection against COVID-19, mean (SD) score	8.8 (2.1)
Air purifiers	
Recommended to others with cancer, mean (SD) score	9.6 (0.9)^b^
Use, No. (%)	
Never	2 (6.5)
A few times, once or twice a week, or with guests	6 (19.4)
Most days or nearly every day	23 (74.2)
Rated in the top 2 of the 4 toolkit components, No. (%)	21 (67.7)
Protection against COVID-19, mean (SD) score	8.5 (2.3)
Rapid tests	
Recommended to others with cancer, mean (SD) score	9.8 (0.6)^b^
No. of tests used (0-5), mean (SD)	1.8 (2.1)
Any use, No. (%)	16 (51.6)
Total No. of tests used among the subset testing (n = 16), mean (SD)	3.6 (1.6)
Rated in the top 2 of the 4 toolkit components, No. (%)	10 (32.3)
Protection against COVID-19, mean (SD) score	8.9 (2.4)

^a^ Includes 31 participants.

^b^ Indicates 1 of the 5 primary questions for assessing acceptability. Scores range from 0 to 10, with scores greater than 7 indicating greater acceptability of the intervention component.

## Discussion

This pilot study demonstrated the feasibility and acceptability of a multilayered COVID-19 mitigation intervention for adults with cancer, meeting the accrual target, achieving 100% retention during follow-up, securing high acceptability ratings, demonstrating high intervention engagement, and yielding a waitlist of 366 patients. Limitations included the sample size, unique historical point, and focus on COVID-19. More research is needed on generalizability and protection against other airborne respiratory viruses (eg, influenza, respiratory syncytial virus) and environmental pathogens. The intervention’s feasibility, acceptability, and modest cost suggest that randomized clinical trials and quality improvement projects are warranted to evaluate effectiveness in reducing respiratory infections and treatment disruptions.

## Notes

Supplement 1. **eMethods.** Conceptual Framework, Aims, Population, Measures, Toolkit, and Statistical Analysis
eReferences

Supplement 2.
Data Sharing Statement
