# Supplementary material for: Feasibility and Acceptability of a Multilayered COVID-19 Mitigation Intervention for Adults With Cancer
Source: JAMA Netw Open. 2026 Feb 24;9(2):e2560547. doi: 10.1001/jamanetworkopen.2025.60547 (PMC12933275; doi:10.1001/jamanetworkopen.2025.60547)
Supplement: Supplement 1. — eMethods. Conceptual Framework, Aims, Population, Measures, Toolkit, and Statistical Analysis eReferences [file jamanetwopen-e2560547-s001.pdf]

Supplementary Online Content

Hoerger M, Pyke N, Zimmerman DE, et al. Feasibility and acceptability of a multilayered COVID-19 mitigation intervention for adults with cancer. *JAMA Netw Open.* 2026;9(2):e2560547. doi:10.1001/jamanetworkopen.2025.60547

eMethods. Conceptual Framework, Aims, Population, Measures, Toolkit, and Statistical Analysis

eReferences

This supplementary material has been provided by the authors to give readers additional information about their work.

© 2026 Hoerger M et al. *JAMA Network Open*.

eMethods. Conceptual Framework, Aims, Population, Measures, Toolkit, and Statistical Analysis

Overview

Patients undergoing cancer therapy remain at high risk of severe outcomes from COVID-19.[1-24] The present investigation involved a pilot intervention trial to evaluate the feasibility and acceptability of a mailed, multi-faceted COVID-19 risk-reduction intervention for U.S. patients undergoing active cancer therapy.

Conceptual Framework

The Swiss Cheese Model or multi-layered mitigation framework is well-suited to managing complex and challenging risk scenarios, such as that of a highly transmissible airborne infectious disease.[5,25-44] The framework acknowledges that each mitigation tool provides a layer of protection but is fallible, or has holes like Swiss cheese. Using multiple layers decreases risk, and this framework is reasonable for reducing COVID-19 risk among oncology patients.[5] Accordingly, we developed a COVID-19 Defense Toolkit for patients undergoing cancer treatment and their families. The toolkit includes a) an educational booklet[45-52] describing airborne transmission,[29-44] how to monitor community levels,[1,2] and evidence underlying the tools that are included in the toolkit or available through one's care team (i.e., vaccines,[6,21,53] pre-exposure prophylaxis,[54-56] and treatments[57,58]), b) high-quality masks[5,22,29,59-65] tailored to patients and families based on size and preference to reduce airborne transmission in community settings, c) air purifiers[39,41,43,66-88] to reduce airborne transmission in the home, and d) rapid antigen tests[89-97] to reduce in-home transmission and facilitate early treatment.

Aims

The study aims were to 1) examine the feasibility of enrolling participants, mailing the toolkits, and retaining participants at 1-month follow up, 2) evaluate the acceptability of the toolkit at follow-up, and 3) estimate intervention costs, with the goal of determining whether to proceed with a randomized clinical trial.

Study Population

Participants were adults with cancer in the U.S. who were undergoing cancer treatment. They enrolled from February to September 2024. Participants were recruited on-site at our cancer center and through earned media coverage via social media, word of mouth, and regional television news. Target enrollment was 30 participants with a maximum of 32 to allow for participants enrolling close in time. An intramural award

© 2026 Hoerger M et al. *JAMA Network Open*.

supported the development of the intervention (Project COVI-CAN), and the American Cancer Society funded most of the pilot data collection (COVI-CAN 2). The research was IRB approved (2023-1540-TU).

Measures

Participants completed consent and eligibility screening forms, a baseline survey (demographics, health history, prior experience and preferences regarding COVID-19 mitigation), and a 1-month follow-up survey. The follow-up survey evaluated intervention utilization behavior and attitudes using quantitative and qualitative questions. Five key questions were used to evaluate acceptability, including the likelihood participants would recommend each of the four intervention components (i.e., booklet, masks, air purifiers, tests) to other patients with cancer, rated on a scale from 0 (not likely to recommend) to 10 (extremely likely to recommend) and how safe the toolkit made them overall on a scale from 0 (not at all safe) to 10 (extremely safe).

COVID-19 Defense Toolkit

The intervention consisted of a mailed, individually-tailored COVID-19 Defense Toolkit designed to reduce the risk of COVID-19 among adults receiving active cancer therapy. Toolkits were shipped in 20 × 18 × 22-inch boxes and contained four evidence-based components: (1) an educational booklet,[45-52] (2) 50 high-filtration masks[5,22,29,59-65] matched to household needs, (3) two air purifiers,[39,41,43,66-88] and (4) five rapid antigen tests.[89-97]

Toolkit Development. Content was informed by our cancer-specific multi-layered mitigation framework[5] and more than 4 years of community engagement that has included leading a national COVID-19 wastewater-surveillance dashboard, local N95 and test-distribution initiatives, hundreds of individual consultations on risk mitigation, 20 small-group stakeholder advisory meetings, and 5 virtual town halls.[2,4,5,22,60,65,98-102] Feedback from patients, caregivers, oncology clinicians, infection-control experts, and industry partners informed toolkit design. Broad efficacy evidence supports each intervention component—psychoeducation,[45-52] masking,[5,22,29,59-65] air purifiers,[39,41,43,66-88] and testing[89-97]—but a clear gap in the literature is real-world effectiveness.[29,48-52,59,65,66,86-88,90,93-97,103-113] Accordingly, the intervention was informed by principles of dissemination and implementation science[10,114] to identify key barriers identified through stakeholder input, such as knowledge gaps, cost, limited access to vetted supplies, and uncertainty about correct use.

© 2026 Hoerger M et al. *JAMA Network Open*.

Educational Booklet. The booklet provided practical evidence-based information on COVID-19 tailored toward helping people with cancer and their families reduce risk of infection and serious outcomes. The 16-page booklet was written at a fifth-grade reading level with optional “deep dive” sidebars with scientific references and statistics for advanced readers. Eight sections summarized (1) excess COVID-19 severity risk in oncology populations; (2) interpreting COVID-19 wastewater surveillance data via national dashboards; (3) airborne transmission; (4) the Swiss Cheese Model of multi-layered mitigation (Figure 1); (5) FDA/CDC-based testing and isolation protocols; (6) respirator selection, fit, and sourcing; (7) home air cleaning strategies; and (8) vaccine, prophylaxis, and treatment options to discuss with oncology teams. Infographics, photographs, and web addresses linking to example products and demonstrations reinforced key messages. We anticipate that a public website based on the booklet will be available by May 1, 2026.

Masks. Participants received 50 masks for personal and family use. All masks were NIOSH-certified N95 respirators or KN95 or KF94 masks with independent testing data demonstrating high filter efficiency, and all were sourced from manufacturers vetted by Project N95. At baseline, participants described household composition, the types of masks they and family used if any, challenges with comfort and sizing, and any preferences surrounding style and fit. An internal algorithm, refined by masking experts from Project N95 and several engineers, translated responses into a customized shipment of 50 masks. The default mask was the 3M Aura, which fits most adults well. Other mask options available included the 3M VFlex (“regular” [very large] or “small” [very small]), Vitacore CAN99 (white or black), Vitacore CAN99e (earloop, available in adult regular or adult small/child), Vitacore CAN95e (white or black), Aegle N95, Powecom KN95, and WellBefore Children’s Petite (XS, small, or regular). Less commonly used options included the Drager X-plore 1950, Harley S-188N, ACI 3120 duckbills, and the strapless flat Readimask (small, large, or XL). Each toolkit contained at least 2-7 models so users could compare fit. Cloth and procedure masks were excluded. The booklet provided instructions for seal checks and links to supplies for reordering.

Air Purifiers. Each participant received two air purifiers. From a dissemination and implementation science perspective,[10,114] we opted to provide participants with pre-assembled versions of do-it-yourself (DIY) or “home build” air purifiers[39,43,58,71-85] that clean the air at 8-12x lower cost[71] than comparable commercially available HEPA devices. Each air purifier is comprised of a standard 20-inch box fan attached to a 4-inch thick

© 2026 Hoerger M et al. *JAMA Network Open*.

20 x 20-inch MERV-13 filter (Tex Air or Nordic Pure) and fitted with a cardboard shroud to improve efficiency. Color options (white/gray [Lasko Classic], black, blue, or purple [Lasko Cool Colors]) allowed aesthetic matching to home décor. Operating on the lowest fan setting, the pair of air purifiers delivered a combined clean air delivery rate of ~600 ft^3/min,[71] which ASHRAE Standard 241[41] recommends for gatherings of up to 10-12 people. This type of air purifier has been widely used in schools with less application elsewhere. The booklet provided placement diagrams, instructions for higher fan settings when a household member may become symptomatic, and links to supplies for reordering filters.

Rapid Antigen Tests. Five FDA-authorized Flowflex tests were provided for participants and their family members. The tests can facilitate early detection to prevent in-home transmission to the patient or help patients to gain early antiviral treatment if positive. The booklet summarized best-practice serial-testing algorithms and emphasized prompt communication of positive results to oncology care teams to enable antiviral therapy. Flowflex was selected for its high accuracy at infectious viral loads (94.7 % sensitivity and 98.2 % specificity for cycle threshold < 25)[89,97,113,115] and low cost among tests of comparable quality.

Toolkit Costs. The cost of the COVID-19 Defense Toolkit was estimated at $218.60 on average. Cost estimates per component were $1.00 for the booklet, $50.00 for the masks (approximately $1.00 on average per mask times 50 masks), $110.60 for the air purifiers ($22.00 per fan, $30.00 per filter, $2.30 for other materials, $1.00 assembly costs, all multiplied by 2 for the pair of air purifiers), $24.00 for the rapid antigen tests ($4.80 per test times 5 tests), and $33 for shipping ($22 postage on average, $9.00 for packaging materials, and $2.00 in labor costs).

Statistical Analyses

A sample size of at least 30 was selected to provide reasonable saturation to test the primary aim of feasibility. The intervention would be deemed feasible if it met all the following conditions: a) enrollment of at least 30 participants, b) successful delivery of at least 90% of intervention toolkits without reshipment, and c) retention of at least 70% of participants at 1-month follow-up. Accrual beyond 30 was expected to provide diminishing marginal returns for troubleshooting product supply chain, packaging, and shipping challenges. Among participants who completed the follow-up, the intervention was evaluated as acceptable if the mean rating for each of the five acceptability items was at least 7 on the 0-10 rating scales. The other quantitative

© 2026 Hoerger M et al. *JAMA Network Open*.

and qualitative questions were used to characterize acceptability in more detail. With the target sample size, 95% CIs provided reasonable precision for determining whether to proceed with a future randomized clinical trial (e.g., 95% CI of ±11% on the 90% proportion, ±14% on the 70% proportion, and ±0.72 points on a 0-10 rating, assuming a SD of <2.00). Tracking utilization data enabled descriptive micro-costing estimates. We assumed that the ongoing costs of each component would be $0.00 to keep using the booklet, $1.00 per mask (with monthly mask utilization expected to be 50-100% of the rate used in the trial, or 75% on average), $30.00 per air purifier filter (2-4 replacement filters per year, or 3 on average), and $5.38 per rapid test at a non-bulk rate; mask and rapid test utilization rates were used to estimate the total ongoing annualized anticipated costs.

© 2026 Hoerger M et al. *JAMA Network Open*.

eReferences

1. Centers for Disease Control and Prevention (CDC) National Wastewater Surveillance System (NWSS). COVID-19 National Wastewater Data: COVID-19 Wastewater Monitoring in the U.S. https://www.cdc.gov/nwss/rv/COVID19-national-data.html. 2025.
2. Hoerger M. Pandemic Mitigation Collaborative - COVID-19 Data Dashboard. http://www.pmc19.com/data. 2025.
3. Meier D, Patkee P, Strange A. The future of excess mortality after COVID-19: The COVID-19 pandemic has been synonymous with excess mortality. *Swiss Re Institute.* 2024.
4. Leston M, Ordóñez-Mena JM, Joy M, et al. The DESTINIES Study: an online Delphi study to build international consensus on the medical conditions and procedures that confer immunosuppression and their respective COVID-19 risk profiles. *EClinicalMedicine.* 2025;83.
5. Hoerger M, Gerhart J, Swartz MC. Variability in COVID-19 vaccine response among people with cancer: what health care strategy best protects the vulnerable? *JAMA oncology.* 2023;9(2):177-179.
6. Lee LY, Tilby M, Starkey T, et al. Association of SARS-CoV-2 spike protein antibody vaccine response with infection severity in patients with cancer: a national COVID cancer cross-sectional evaluation. *JAMA oncology.* 2023;9(2):188-196.
7. Gong IY, Vijenthira A, Powis M, et al. Association of COVID-19 vaccination with breakthrough infections and complications in patients with cancer. *JAMA oncology.* 2023;9(3):386-394.
8. Potter AL, Vaddaraju V, Venkateswaran S, et al. Deaths due to COVID-19 in patients with cancer during different waves of the pandemic in the US. *JAMA oncology.* 2023;9(10):1417-1422.
9. Pinato DJ, Tabernero J, Bower M, et al. Prevalence and impact of COVID-19 sequelae on treatment and survival of patients with cancer who recovered from SARS-CoV-2 infection: evidence from the OnCovid retrospective, multicentre registry study. *The Lancet Oncology.* 2021;22(12):1669-1680.
10. Dagher H, Chaftari A-M, Subbiah IM, et al. Long COVID in cancer patients: preponderance of symptoms in majority of patients over long time period. *Elife.* 2023;12.
11. Monroy-Iglesias MJ, Tremble K, Russell B, et al. Long-term effects of COVID-19 on cancer patients: the experience from Guy's Cancer Centre. *Future Oncology.* 2022;18(32):3585-3594.

© 2026 Hoerger M et al. *JAMA Network Open*.

12. Ofer J, Drozdinsky G, Basharim B, Turjeman A, Eliakim-Raz N, Stemmer SM. Mortality and Hospitalization Risks in Patients With Cancer and the SARS-CoV-2 Omicron Variant. *JAMA oncology.* 2024;10(1):137-138.

13. Turtle L, Elliot S, Drake TM, et al. Changes in hospital mortality in patients with cancer during the COVID-19 pandemic (ISARIC-CCP-UK): a prospective, multicentre cohort study. *The Lancet Oncology.* 2024.

14. Centers for Disease Control and Prevention (CDC). People with Certain Medical Conditions. https://www.cdc.gov/coronavirus/2019-ncov/need-extra-precautions/people-with-medical-conditions.html#MedicalConditionsAdults. 2024.

15. Centers for Disease Control and Prevention (CDC). Cancer and COVID-19. https://www.cdc.gov/cancer/dcpc/about/covid-19.htm. 2023.

16. Centers for Disease Control and Prevention (CDC). COVID-19 and Cancer Deaths. https://www.cdc.gov/cancer/dcpc/research/articles/covid-19-cancer-deaths.html. 2022.

17. National Cancer Institute (NCI). COVID-19: What People with Cancer Should Know. https://www.cancer.gov/about-cancer/coronavirus/coronavirus-cancer-patient-information. 2024.

18. American Cancer Society (ACS). Infections in People with Cancer. https://www.cancer.org/cancer/managing-cancer/side-effects/infections.html. 2024.

19. Robineau O, Zins M, Touvier M, et al. Long-lasting symptoms after an acute COVID-19 infection and factors associated with their resolution. *JAMA Network Open.* 2022;5(11):e2240985-e2240985.

20. Llanos AA, Ashrafi A, Ghosh N, et al. Evaluation of inequities in cancer treatment delay or discontinuation following SARS-CoV-2 infection. *JAMA Network Open.* 2023;6(1):e2251165-e2251165.

21. Rini BI, Best AF, Bowman MD, et al. Risk Factors for COVID-19–Related Hospitalization and Death in Patients With Cancer: The National Cancer Institute COVID-19 in Cancer Patients Study (NCCAPS). *JAMA oncology.* 2025.

22. Hoerger M, Rivera D, Mossman B, Sherard B, Peyser T, Alcorn TM. Masking Policies at National Cancer Institute–Designated Cancer Centers During Winter 2023 to 2024 COVID-19 Surge. *JAMA Network Open.* 2024;7(7):e2424999-e2424999.

© 2026 Hoerger M et al. *JAMA Network Open*.

23. Han A. Masks in the Clinical Center Past/Present/Future. https://www.ccrhb.od.nih.gov/presentations/10182024/3.Han_Masks%20in%20CC_CCRHB_10_18_24.pdf. 2024 (Oct 18).

24. Pak TR, Chen T, Kanjilal S, McKenna CS, Rhee C, Klompas M. Testing and masking policies and hospital-onset respiratory viral infections. *JAMA Network Open.* 2024;7(11):e2448063-e2448063.

25. Roberts S. The Swiss Cheese Model of Pandemic Defense. *New York Times*2020.

26. Ngo T. To slow the spread of COVID-19, we need to bring back the Swiss Cheese Model of pandemic response. *Health Affairs Forefront.* 2021.

27. Escandón K, Rasmussen AL, Bogoch II, et al. COVID-19 false dichotomies and a comprehensive review of the evidence regarding public health, COVID-19 symptomatology, SARS-CoV-2 transmission, mask wearing, and reinfection. *BMC infectious diseases.* 2021;21(1):1-47.

28. Shabani T, Jerie S, Shabani T. A comprehensive review of the Swiss cheese model in risk management. *Safety in Extreme Environments.* 2024;6(1):43-57.

29. Greenhalgh T, MacIntyre C, Baker M, al. e. Masks and Respirators for Prevention of Respiratory Infections: A State of the Science Review. *Microbiology Reviews.* 2024.

30. Cheng P, Luo K, Xiao S, et al. Predominant airborne transmission and insignificant fomite transmission of SARS-CoV-2 in a two-bus COVID-19 outbreak originating from the same pre-symptomatic index case. *Journal of hazardous materials.* 2022;425:128051.

31. Greenhalgh T, Jimenez JL, Prather KA, Tufekci Z, Fisman D, Schooley R. Ten scientific reasons in support of airborne transmission of SARS-CoV-2. *The lancet.* 2021;397(10285):1603-1605.

32. Jimenez JL, Marr LC, Randall K, et al. What were the historical reasons for the resistance to recognizing airborne transmission during the COVID-19 pandemic? *Indoor Air.* 2022;32(8):e13070.

33. Lewis D. Why the WHO took two years to say COVID is airborne. *Nature.* 2022;604(7904):26-31.

34. Samet JM, Prather K, Benjamin G, et al. Airborne transmission of severe acute respiratory syndrome coronavirus 2 (SARS-CoV-2): what we know. *Clinical Infectious Diseases.* 2021;73(10):1924-1926.

© 2026 Hoerger M et al. *JAMA Network Open*.

35. Tang JW, Bahnfleth WP, Bluyssen PM, et al. Dismantling myths on the airborne transmission of severe acute respiratory syndrome coronavirus-2 (SARS-CoV-2). *Journal of Hospital Infection.* 2021;110:89-96.

36. Wang CC, Prather KA, Sznitman J, et al. Airborne transmission of respiratory viruses. *Science.* 2021;373(6558):eabd9149.

37. Pöhlker ML, Pöhlker C, Krüger OO, et al. Respiratory aerosols and droplets in the transmission of infectious diseases. *Reviews of Modern Physics.* 2023;95(4):045001.

38. Bahl P, Doolan C, De Silva C, Chughtai AA, Bourouiba L, MacIntyre CR. Airborne or droplet precautions for health workers treating coronavirus disease 2019? *The Journal of infectious diseases.* 2022;225(9):1561-1568.

39. Centers for Disease Control and Prevention (CDC). Ventilation in Buildings: Summary of Recent Changes. https://www.cdc.gov/coronavirus/2019-ncov/community/ventilation.html. 2023.

40. World Health Organization. Global technical consultation report on proposed terminology for pathogens that transmit through the air. https://cdn.who.int/media/docs/default-source/documents/emergencies/global-technical-consultation-report-on-proposed-terminology-for-pathogens-that-transmit-through-the-air.pdf. 2024.

41. American Society of Heating, Refrigerating and Air-Conditioning Engineers (ASHRAE). Standard 241-2023, Control of Infectious Aerosols. https://www.ashrae.org/technical-resources/standards-and-guidelines/read-only-versions-of-ashrae-standards. 2023.

42. National Academies of Sciences E, and Medicine,. Health Risks of Indoor Exposure to Fine Particulate Matter and Practical Mitigation Solutions (2024). http://nap.nationalacademies.org/27341. 2024.

43. The White House. Let's Clear The Air On COVID. https://www.whitehouse.gov/ostp/news-updates/2022/03/23/lets-clear-the-air-on-covid/. 2022.

44. The White House. Press Briefing by Press Secretary Karine Jean-Pierre and COVID-19 Response Coordinator Dr. Ashish Jha. https://www.whitehouse.gov/briefing-room/statements-releases/2022/10/25/press-briefing-by-press-secretary-karine-jean-pierre-and-covid-19-response-coordinator-dr-ashish-jha-6/. 2022.

© 2026 Hoerger M et al. *JAMA Network Open*.

45. Berkman ND, Sheridan SL, Donahue KE, et al. Health literacy interventions and outcomes: an updated systematic review. *Evidence report/technology assessment.* 2011(199):1-941.

46. Stacey D, Samant R, Bennett C. Decision making in oncology: a review of patient decision aids to support patient participation. *CA: a cancer journal for clinicians.* 2008;58(5):293-304.

47. Simonsmeier BA, Flaig M, Simacek T, Schneider M. What sixty years of research says about the effectiveness of patient education on health: a second order meta-analysis. *Health Psychology Review.* 2022;16(3):450-474.

48. Takagi MA, Hess S, Smith Z, et al. The impact of educational interventions on COVID-19 and vaccination attitudes among patients in Michigan: A prospective study. *Frontiers in Public Health.* 2023;11:1144659.

49. Mistree D, Loyalka P, Fairlie R, et al. Instructional interventions for improving COVID-19 knowledge, attitudes, behaviors: Evidence from a large-scale RCT in India. *Social Science & Medicine.* 2021;276:113846.

50. Anda SD, Budd EL, Halvorson S, et al. Effects of a health education intervention for COVID-19 prevention in Latinx communities: a cluster-randomized controlled trial. *American Journal of Public Health.* 2022;112(S9):S923-S927.

51. Hoerger MPM. Teaching a Graduate-level Research Methodology Course with Comprehensive COVID-19 Precautions: Implications for Safety Knowledge, Attitudes, Behavior, and Inclusivity. 2023.

52. Gobbo EL, Hanson C, Abunnaja KS, van Wees SH. Do peer-based education interventions effectively improve vaccination acceptance? a systematic review. *BMC Public Health.* 2023;23(1):1354.

53. Skarbinski J, Elkin EP, Ziemba YC, et al. COVID-19 vaccine booster uptake and effectiveness among US adults with cancer. *JAMA oncology.* 2025.

54. Haidar G, Thomas S, Loubet P, et al. Efficacy and safety of sipavibart for prevention of COVID-19 in individuals who are immunocompromised (SUPERNOVA): a randomised, controlled, double-blind, phase 3 trial. *The Lancet infectious diseases.* 2025;25(7):813-826.

© 2026 Hoerger M et al. *JAMA Network Open*.

55. Wolfe CR, Cohen J, Mahoney K, et al. Safety and Efficacy of Pemivibart, a Long-Acting Monoclonal Antibody, for Prevention of Symptomatic COVID-19: Interim Results From a Phase 3 Randomized Clinical Trial (CANOPY). *Clinical Infectious Diseases.* 2025:ciaf265.
56. Bock A. New guidance helps clinicians use pemivibart to protect immunocompromised patients from COVID-19. *JAMA.* 2024;332(14):1127-1129.
57. Patel P, Wentworth DE, Daskalakis D. COVID-19 therapeutics for nonhospitalized older adults. *JAMA.* 2024;332(18):1511-1512.
58. Raglow Z, Malani PN, Petty LA. Outpatient treatment for COVID-19. *JAMA.* 2023;330(13):1295-1295.
59. Rashid TU, Sharmeen S, Biswas S. Effectiveness of N95 masks against SARS-CoV-2: performance efficiency, concerns, and future directions. *ACS Chemical Health & Safety.* 2022;29(2):135-164.
60. Hoerger M, Gerhart J, Swartz MC. Evidence Base for Health Care Strategies to Protect Vulnerable Patients During the COVID-19 Pandemic—Reply. *JAMA Oncology.* 2023(9):1150-1151.
61. Centers for Disease Control and Prevention (CDC). Respirator Approval Information: How to apply for NIOSH Respirator Approval. https://www.cdc.gov/niosh/npptl/RespApprovalInfo.html. 2023.
62. Sickbert-Bennett EE, Samet JM, Clapp PW, et al. Filtration efficiency of hospital face mask alternatives available for use during the COVID-19 pandemic. *JAMA Internal Medicine.* 2020;180(12):1607-1612.
63. Lindsley WG, Blachere FM, Law BF, Beezhold DH, Noti JD. Efficacy of face masks, neck gaiters and face shields for reducing the expulsion of simulated cough-generated aerosols. *Aerosol Science and Technology.* 2021;55(4):449-457.
64. Tcharkhtchi A, Abbasnezhad N, Seydani MZ, Zirak N, Farzaneh S, Shirinbayan M. An overview of filtration efficiency through the masks: Mechanisms of the aerosols penetration. *Bioactive materials.* 2021;6(1):106-122.
65. Moran JB, Dunn A, Kim S, Zapolin D, Rivera D, Hoerger M. Community-based N95 distribution during the COVID-19 Omicron BA. 1 surge: feasibility, 1-month utilization, and price implications. *Translational Behavioral Medicine.* 2023:ibad019.

© 2026 Hoerger M et al. *JAMA Network Open*.

66. Baker JM, Nakayama JY, O'Hegarty M. CDC: SARS-CoV-2 B. 1.1. 529 (Omicron) variant transmission within households—four US jurisdictions, November 2021–February 2022. MMWR. Morbidity and mortality weekly report Web site. https://www.cdc.gov/mmwr/volumes/71/wr/mm7109e1.htm. 2022.

67. Ueki H, Ujie M, Komori Y, Kato T, Imai M, Kawaoka Y. Effectiveness of HEPA filters at removing infectious SARS-CoV-2 from the air. *Msphere.* 2022;7(4):e00086-00022.

68. Zhang S, Stamp S, Cooper E, Curran K, Mumovic D. Evaluating the impact of air purifiers and window operation upon indoor air quality-UK nurseries during Covid-19. *Building and Environment.* 2023;243:110636.

69. Liu DT, Phillips KM, Speth MM, Besser G, Mueller CA, Sedaghat AR. Portable HEPA purifiers to eliminate airborne SARS-CoV-2: a systematic review. *Otolaryngology–Head and Neck Surgery.* 2022;166(4):615-622.

70. Christopherson DA, Yao WC, Lu M, Vijayakumar R, Sedaghat AR. High-efficiency particulate air filters in the era of COVID-19: function and efficacy. *Otolaryngology–Head and Neck Surgery.* 2020;163(6):1153-1155.

71. Srikrishna D. Can 10× cheaper, lower-efficiency particulate air filters and box fans complement High-Efficiency Particulate Air (HEPA) purifiers to help control the COVID-19 pandemic? *Science of the Total Environment.* 2022;838:155884.

72. Wilke C. ACS Publications: A Conversation with Richard Corsi. https://doi.org/10.1021/acscentsci.2c00500. 2022.

73. Wikipedia. Corsi–Rosenthal Box. https://en.wikipedia.org/wiki/Corsi%E2%80%93Rosenthal_Box. 2024.

74. Dal Porto R, Kunz MN, Pistochini T, Corsi RL, Cappa CD. Characterizing the performance of a do-it-yourself (DIY) box fan air filter. *Aerosol Science and Technology.* 2022;56(6):564-572.

75. He R, Liu W, Elson J, Vogt R, Maranville C, Hong J. Airborne transmission of COVID-19 and mitigation using box fan air cleaners in a poorly ventilated classroom. *Physics of Fluids.* 2021;33(5).

76. Manz KE, Dodson RE, Liu Y, et al. Effects of Corsi-Rosenthal boxes on indoor air contaminants: non-targeted analysis using high resolution mass spectrometry. *Journal of Exposure Science & Environmental Epidemiology.* 2023;33(4):537-547.

© 2026 Hoerger M et al. *JAMA Network Open*.

77. Vartiainen VA, Hela J, Luoto A, et al. The effect of room air cleaners on infection control in day care centres. *Indoor Environments.* 2024;1(1):100007.

78. Alvarenga M, Dias J, Lima B, Gomes A, Monteiro G. The implementation of portable air-cleaning technologies in healthcare settings–a scoping review. *Journal of Hospital Infection.* 2023;132:93-103.

79. Woods L. UConn Gifts Clean Air: 150 DIY Indoor Air Filters Distributed to Connecticut Community for Protection from Wildfire Air Particles. https://today.uconn.edu/2023/06/uconn-gifts-clean-air-over-100-d-i-y-indoor-air-filters-distributed-to-connecticut-community-for-protection-from-wildfire-air-particles/. 2023.

80. Hannah D. Smithsonian Magazine: The Homemade Air Purifier That's Been Saving Lives During the Covid-19 Pandemic. https://www.smithsonianmag.com/innovation/homemade-air-purifier-thats-been-saving-lives-during-covid-19-pandemic-180979681/. 2022.

81. Meininger K. The Brown Daily Herald: School of Public Health installs Cube air cleaners as additional COVID-19 prevention measure. 2021.

82. Filmer S. ASU News: ASU volunteers build DIY air filters to help Valley schools fight COVID-19. https://news.asu.edu/20220126-arizona-impact-asu-students-faculty-build-filter-boxes-schools-fight-covid-19. 2022.

83. United States Environmental Protection Agency (EPA). Air Cleaners, HVAC Filters, and Coronavirus (COVID-19). https://www.epa.gov/coronavirus/air-cleaners-hvac-filters-and-coronavirus-covid-19. 2024.

84. Woods L. UConn Today: EPA Testing Shows the Power of DIY Air Filters to Trap Viruses. https://today.uconn.edu/2023/10/epa-testing-shows-the-power-of-d-i-y-air-filters-to-trap-viruses/. 2023.

85. 3M News Center. 3M scientists: This Corsi-Rosenthal box movement is legit. https://news.3m.com/2022-02-24-3M-scientists-This-Corsi-Rosenthal-box-movement-is-legit. 2022.

86. Ebrahimifakhar A, Poursadegh M, Hu Y, Yuill DP, Luo Y. A systematic review and meta-analysis of field studies of portable air cleaners: Performance, user behavior, and by-product emissions. *Science of The Total Environment.* 2023:168786.

87. Morris AC, Sharrocks K, Bousfield R, et al. The removal of airborne severe acute respiratory syndrome coronavirus 2 (SARS-CoV-2) and other microbial bioaerosols by air filtration on coronavirus disease

© 2026 Hoerger M et al. *JAMA Network Open*.

2019 (COVID-19) surge units. *Clinical Infectious Diseases: An Official Publication of the Infectious Diseases Society of America.* 2022;75(1):e97.

88. Dodson RE, Manz KE, Burks SR, et al. Does using Corsi–Rosenthal boxes to mitigate COVID-19 transmission also reduce indoor air concentrations of PFAS and phthalates? *Environmental Science & Technology.* 2022;57(1):415-427.

89. Dinnes J, Sharma P, Berhane S, et al. Rapid, point-of-care antigen tests for diagnosis of SARS-CoV-2 infection. *Cochrane database of systematic reviews.* 2022(7).

90. Mina MJ, Peto TE, García-Fiñana M, Semple MG, Buchan IE. Clarifying the evidence on SARS-CoV-2 antigen rapid tests in public health responses to COVID-19. *The Lancet.* 2021;397(10283):1425-1427.

91. Lee E. At-Home COVID-19 Antigen Test Kits: Where to Buy and What You Should Know. New York Times. https://www.nytimes.com/wirecutter/reviews/at-home-covid-test-kits/. 2023.

92. Collins F, Adam S, Colvis C, et al. The NIH-led research response to COVID-19. *Science.* 2023;379(6631):441-444.

93. Todsen T, Jakobsen KK, Grønlund MP, et al. COVID-19 Rapid Antigen Tests With Self-Collected vs Health Care Worker–Collected Nasal and Throat Swab Specimens: A Randomized Clinical Trial. *JAMA network open.* 2023;6(12):e2344295-e2344295.

94. Viloria Winnett A, Akana R, Shelby N, et al. Daily SARS-CoV-2 Nasal Antigen Tests Miss Infected and Presumably Infectious People Due to Viral Load Differences among Specimen Types. *Microbiology Spectrum.* 2023:e01295-01223.

95. Regev-Yochay G, Kriger O, Mina MJ, et al. Real world performance of SARS-CoV-2 antigen rapid diagnostic tests in various clinical settings. *Infection Control & Hospital Epidemiology.* 2022:1-20.

96. Katzenschlager S, Brümmer LE, Schmitz S, et al. Comparing SARS-CoV-2 antigen-detection rapid diagnostic tests for COVID-19 self-testing/self-sampling with molecular and professional-use tests: a systematic review and meta-analysis. *Scientific Reports.* 2023;13(1):21913.

97. Byrne RL, Aljayyoussi G, Greenland-Bews C, et al. Comparison of the analytical and clinical sensitivity of thirty-four rapid antigen tests with the most prevalent SARS-CoV-2 variants of concern during the COVID-19 pandemic in the UK. *medRxiv.* 2023:2023.2007. 2024.23293072.

© 2026 Hoerger M et al. *JAMA Network Open*.

98. Hoerger M, Kim S, Mossman B, et al. Cultivating community-based participatory research (CBPR) to respond to the COVID-19 pandemic: an illustrative example of partnership and topic prioritization in the food services industry. *BMC Public Health.* 2023;23(1):1939.

99. Hoerger M, Alonzi S, Mossman B. Scenario planning: a framework for mitigating uncertainty in implementing strategic behavioral medicine initiatives during the COVID-19 pandemic. *Translational Behavioral Medicine.* 2022;12(3):486-488.

100. Hoerger M. Public Health's" Forgotten Children" of the COVID-19 Pandemic: Creating the Next Generation of Leaders in Serious Illness Care. *Journal of Palliative Medicine.* 2024;27(4).

101. Cruz P, Lam JM, Abdalla J, et al. Immunobridging trials: an important tool to protect vulnerable and immunocompromised patients against evolving pathogens. *Vaccines.* 2024;13(1):19.

102. Davis L, Higgs M, Snaith A, et al. Dysregulation of lipid metabolism, energy production, and oxidative stress in myalgic encephalomyelitis/chronic fatigue syndrome, Gulf War Syndrome and fibromyalgia. *Frontiers in Neuroscience.* 2025;19:1498981.

103. Jefferson T, Dooley L, Ferroni E, et al. Physical interventions to interrupt or reduce the spread of respiratory viruses. *Cochrane database of systematic reviews.* 2023(1).

104. Wang RC, Degesys NF, Fahimi J, et al. Incidence of Fit Test Failure During N95 Respirator Reuse and Extended Use. *JAMA Network Open.* 2024;7(1):e2353631-e2353631.

105. Falkenberg T, Wasser F, Zacharias N, Mutters N, Kistemann T. Effect of portable HEPA filters on COVID-19 period prevalence: an observational quasi-interventional study in German kindergartens. *BMJ open.* 2023;13(7).

106. Haslam A, Prasad V. Evidence Base for Health Care Strategies to Protect Vulnerable Patients During the COVID-19 Pandemic. *JAMA oncology.* 2023;9(8):1150-1150.

107. Thampi N, Schwartz KL, Brown KA. School-Based Mandatory Masking Policies and Absenteeism in Ottawa, Canada, in 2022. *JAMA Network Open.* 2023;6(7):e2325799-e2325799.

108. Cowger TL, Murray EJ, Clarke J, et al. Lifting universal masking in schools—COVID-19 incidence among students and staff. *New England Journal of Medicine.* 2022;387(21):1935-1946.

© 2026 Hoerger M et al. *JAMA Network Open*.

109. Soares-Weiser K. Statement on ‘Physical interventions to interrupt or reduce the spread of respiratory viruses’ review. *Cochrane.* 2023.

110. Gurbaxani BM, Hill AN, Patel P. Unpacking cochrane’s update on masks and COVID-19. In*.* Vol 113: American Public Health Association; 2023:1074-1078.

111. Abaluck J, Kwong LH, Styczynski A, et al. Impact of community masking on COVID-19: a cluster-randomized trial in Bangladesh. *Science.* 2022;375(6577):eabi9069.

112. Abdul-Mutakabbir JC, Tillman III F, Marcelin JR, Saunders IM, Arya V. Slowed progression: the utility of Test to Treat initiatives in improving the neglected inequities of COVID-19 among racially/ethnically minoritized groups. *Journal of the American Pharmacists Association.* 2023;63(1):424-429.

113. Smith DG. Do Rapid Tests Still Work? International New York Times. https://www.nytimes.com/2023/01/25/well/mind/covid-rapid-tests.html. 2023.

114. Rabin BA, Cakici J, Golden CA, Estabrooks PA, Glasgow RE, Gaglio B. A citation analysis and scoping systematic review of the operationalization of the Practical, Robust Implementation and Sustainability Model (PRISM). *Implementation Science.* 2022;17(1):62.

115. Abusrewil Z, Alhudiri IM, Kaal HH, et al. Time scale performance of rapid antigen testing for SARS-CoV-2: Evaluation of 10 rapid antigen assays. *Journal of Medical Virology.* 2021;93(12):6512-6518.

© 2026 Hoerger M et al. *JAMA Network Open*.
