# Supplementary material for: Feasibility and Acceptability of a Multilayered COVID-19 Mitigation Intervention for Adults With Cancer
Source: JAMA Netw Open. 2026 Feb 24;9(2):e2560547. doi: 10.1001/jamanetworkopen.2025.60547 (PMC12933275; doi:10.1001/jamanetworkopen.2025.60547)
Supplement: Supplement 2. — Data Sharing Statement [file jamanetwopen-e2560547-s002.pdf]

Data Sharing Statement

Hoerger. Feasibility and Acceptability of a Multilayered COVID-19 Mitigation Intervention for Adults With Cancer. *JAMA Netw Open*. Published February 24, 2026.
doi:10.1001/jamanetworkopen.2025.60547

Data
Data available: No

Additional Information
Explanation for why data not available: Public sharing is not possible due to the sensitivity of the data. Deidentified limited datasets can be shared upon reasonable request.
